# Editorial: Myocardial Remodeling: Mechanisms and Translational Implications

**DOI:** 10.3389/fphar.2022.930387

**Published:** 2022-05-26

**Authors:** Jerome Roncalli, Hélène Tronchère, Antonio Lax, Oxana Kunduzova

**Affiliations:** ^1^ Department of Cardiology, Institute CARDIOMET, University Hospital of Toulouse, Toulouse, France; ^2^ INSERM I2MC - UMR1297, Toulouse, France; ^3^ ICTC Research Group/Heart Failure Health Science Campus, University of Murcia, Murcia, Spain

**Keywords:** renin-angiotensin-aldosterone system, myocardial remodeling, hypertrophy, inflammation, fibrosis

Left ventricular remodeling is an adaptive process modifying the ventricular size, shape, structure and mass of the myocardium. The ensuing cardiomyocytes loss, thereby activate intracellular signaling pathways, inflammatory reaction, and stimulate the renin-angiotensin-aldosterone (RAAS) and sympathetic nervous systems (Pfeffer et al., 1990; Rouleau et al., 1993). All of these result in myocardial fibrosis formation and ventricular cavity dilation (Liu et al., 2012; Van Berlo et al., 2013; Xie et al., 2013). Left ventricular remodeling remains the major determinant of cardiac function and survival after recovery from acute myocardial infarction (AMI) (White et al., 1987). Different available and promising therapeutic approaches are available to treat and attenuate the adverse effects of cardiac remodeling process by targeting the underlying pathophysiological mechanisms. Mainly, the RAAS regulates post-AMI cardiac remodeling process (Belge et al., 2014). As a consequence, blocking RAAS acts in a manner to stop left ventricular remodeling, reduce mortality and improve short- and long-term survival (Pfeffer et al., 1990). The goal of this Research Topic was to understand how aberrant cardiac remodeling contributes to the development and progression of heart failure (HF) and how to exploit this knowledge for therapeutic benefits to improve cardiac function and to prevent HF progression. In this Research Topic we have eight original research articles and one review article summarizing the recent advances in myocardial remodeling.

In their study, Zhang et al. intend to investigate the role and mechanisms of Sestrin2 (Sesn2), a stress-induced protein, in cardiac hypertrophy with the use of Sesn2 transgenic and AMPKα2 knockout mice by establishing a pressure overload-induced cardiac hypertrophy model via aortic banding surgery. Regarding cardiac hypertrophy in women, Wang et al. investigated the effects of G protein–coupled estrogen receptor 30 (GPR30), a membrane receptor of estrogen that displays protective roles in diverse cardiovascular diseases and investigated the effects of GPR30, activation on transverse aortic constriction (TAC)-induced cardiac hypertrophy of aged female mice. The novel finding of this study was that GPR30 activation could reduce TAC-induced cardiac fibrosis through downregulation of the MMP-9 level, which may provide the potential therapeutic targets for the treatment of pathological cardiac hypertrophy in postmenopausal women.

Mitochondrial dysfunction also plays an important role in the pathology of cardiac hypertrophy. Findings by Chen et al. suggested that quercetin, a natural flavonol agent, protected mitochondrial function by modulating SIRT3/PARP-1 pathway, contributing to the inhibition of cardiac hypertrophy in spontaneously hypertensive rats (SHRs) and H9c2 cells.

Then, atrial fibrillation (AF) which is the most common sustained cardiac arrhythmia in clinical setting, is associated with metabolic disorder, especially defective fatty acids oxidation (FAO). Thus, promoting FAO could prevent AF occurrence. Zhang et al. demonstrated that FAO promotion via L-carnitine attenuated obesity-mediated AF and structural remodeling by activating AMP-activated protein kinase (AMPK) signaling and alleviating atrial lipotoxicity. RAAS inhibitors can also inhibited the occurrence and development of AF induced by atrial fibrosis and realized significant benefits for the long-term survival of AF patients (Han et al., 2013; Turin et al., 2018). However, these conventional drugs cannot completely cure atrial fibrosis (McDonagh et al., 2021). Therefore, Hu et al. investigated the mechanisms and interventions of atrial fibrosis to reduce the atrial structural remodeling and electrical remodeling caused by atrial fibrosis in order to reduce the occurrence and development of AF. Jarkovská et al. show that effective suppression of electrical proarrhythmic remodeling and mortality but not hypertrophy indicates that the beneficial therapeutic effects of ACE inhibitor trandolapril in volume overload heart failure might be dissociated from pure antihypertrophic effects.

Moreover, mineralocorticoid receptor antagonists (MRA) have been described to reduce reactive fibrosis and improve cardiac function. However, Demkes et al. failed to demonstrate that combined treatment with GLP-1R agonist exenatide and MRA potassium canrenoate could minimize cardiac injury and limit progression to chronic HF in a pig model of ischemic/reperfusion.

Recently, LCZ696 (valsartan/sacubitril), the first of the new ARNI (angiotensin receptor-neprilysin inhibitor) drug class, has been recently approved for the treatment of chronic HF patients with reduced ejection fraction (HFrEF) after the PARADIGM- HF trial (McMurray et al., 2014; Campbell, 2017). The addition of the neprilysin component in LCZ696 augments plasma levels of natriuretic peptides (Voors et al., 2013) that counteract the RAAS and promote vasodilation, natriuresis, and inhibit fibrosis and hypertrophy. Despite recent formal recognition of ARNI by guideline authorities, there is a striking paucity of mechanistic data on the effect of ARNI on cardiac remodeling (McDonagh et al., 2021). In this Research Topic, Liu et al., investigated the mechanisms underlying the cardioprotective action of ARNI in the context of fibrosis and remodeling after AMI that are mostly unknown. They found that the improvement of cardiac function in the ARNI group was more significant than a single RAAS blocker. Then, they investigated the mechanisms involved in the role of the Wnt/β-catenin axis in the prevention of myocardial fibrosis and improvement of myocardial remodeling in the context of ARNI treatment (Palevski et al., 2017; Fu et al., 2019). Nevertheless, in the PARADISE-MI trial LCZ696 was not associated with a significant lower incidence of death from cardiovascular causes or incident HF compared to ramipril alone after an AMI (Pfeffer et al., 2021). At this stage, it is difficult to conclude, but the interesting findings shown by Liu et al., help to learn how ARNI acts and we will probably learn more from ongoing studies in this area.

Overall, the traditional guideline directed therapies target the RAAS and the sympathetic nervous system, but recently, cyclic guanosine 3′,5′-monophosphate (cGMP) and its downstream protein kinase G (PKG) signaling has attracted attention as a novel therapeutic target (Tsai and Kass, 2009). cGMP is a second messenger regulated through natriuretic peptide and nitric oxide pathways. In their review, Numata and Takimoto highlighted preclinical evidence of the benefits of cGMP/PKG augmentation in HF models.

In conclusion, this Research Topic provides an overview of the novel mechanisms and translational implications involved in myocardial remodeling leading to HF. This field of research is evolving rapidly, and it ultimately holds promise for more reliable and sensitive development of novel treatments.

## References

[B1] Belge C. Hammond J. Dubois-Deruy E. Manoury B. Hamelet J. Beauloye C. (2014). Enhanced Expression of β3-adrenoceptors in Cardiac Myocytes Attenuates Neurohormone-Induced Hypertrophic Remodeling through Nitric Oxide Synthase. Circulation. 129 (4), 451–462. 10.1161/CIRCULATIONAHA.113.004940 24190960

[B2] Campbell D. J. (2017). Long-term Neprilysin Inhibition - Implications for ARNIs. Nat. Rev. Cardiol. 14 (3), 171–186. 10.1038/nrcardio.2016.200 27974807

[B3] Fu W. B. Wang W. E. Zeng C. Y. (2019). Wnt Signaling Pathways in Myocardial Infarction and the Therapeutic Effects of Wnt Pathway Inhibitors. Acta Pharmacol. Sin. 40 (1), 9–12. 10.1038/s41401-018-0060-4 30002488 PMC6318317

[B4] Han M. Zhang Y. Sun S. Wang Z. Wang J. Xie X. (2013). Renin-angiotensin System Inhibitors Prevent the Recurrence of Atrial Fibrillation: a Meta-Analysis of Randomized Controlled Trials. J. Cardiovasc. Pharmacol. 62 (4), 405–415. 10.1097/FJC.0b013e3182a094a1 23921300

[B5] Liu Q. Chen Y. Auger-Messier M. Molkentin J. D. (2012). Interaction between NFκB and NFAT Coordinates Cardiac Hypertrophy and Pathological Remodeling. Circ. Res. 110 (8), 1077–1086. 10.1161/CIRCRESAHA.111.260729 22403241 PMC3341669

[B6] McDonagh T. A. Metra M. Adamo M. Gardner R. S. Baumbach A. Böhm M. (2021). 2021 ESC Guidelines for the Diagnosis and Treatment of Acute and Chronic Heart Failure. Eur. Heart J. 42 (36), 3599–3726. 10.1093/eurheartj/ehab368 34447992

[B7] McMurray J. J. Packer M. Desai A. S. Gong J. Lefkowitz M. P. Rizkala A. R. (2014). Angiotensin-Neprilysin Inhibition versus Enalapril in Heart Failure. N. Engl. J. Med. 371 (11), 993–1004. 10.1056/nejmoa1409077 25176015

[B8] Pfeffer M. A. Braunwald E. Moyé L. A. Basta L. Brown E. J. Jr Cuddy T. E. (1990). Effect of Captopril on Mortality and Morbidity in Patients with Left Ventricular Dysfunction after Myocardial Infarction. Results of the Survival and Ventricular Enlargement Trial. The SAVE Investigators. N. Engl. J. Med. 327 (10), 669–677. 10.1056/NEJM199209033271001 1386652

[B9] Pfeffer M. A. Braunwald E. (1990). Ventricular Remodeling after Myocardial Infarction. Experimental Observations and Clinical Implications. Circulation. 81 (4), 1161–1172. 10.1161/01.cir.81.4.1161 2138525

[B10] Pfeffer M. A. Claggett B. Lewis E. F. Granger C. B. Køber L. Maggioni A. P. (2021). Angiotensin Receptor-Neprilysin Inhibition in Acute Myocardial Infarction. N. Engl. J. Med. 385 (20), 1845–1855. 10.1056/NEJMoa2104508 34758252

[B11] Rouleau J. L. De Champlain J. Klein M. Bichet D. Moyé L. Packer M. (1993). Activation of Neurohumoral Systems in Postinfarction Left Ventricular Dysfunction. J. Am. Coll. Cardiol. 22 (2), 390–398. 10.1016/0735-1097(93)90042-y 8101532

[B12] Tsai E. J. Kass D. A. (2009). Cyclic GMP Signaling in Cardiovascular Pathophysiology and Therapeutics. Pharmacol. Ther. 122 (3), 216–238. 10.1016/j.pharmthera.2009.02.009 19306895 PMC2709600

[B13] Turin A. Bax J. J. Doukas D. Joyce C. Lopez J. J. Mathew V. (2018). Interactions Among Vitamin D, Atrial Fibrillation, and the Renin-Angiotensin-Aldosterone System. Am. J. Cardiol. 122 (5), 780–784. 10.1016/j.amjcard.2018.05.013 30057228

[B14] Van Berlo J. H. Maillet M. Molkentin J. D. (2013). Signaling Effectors Underlying Pathologic Growth and Remodeling of the Heart. J. Clin. Invest. 123 (1), 37–45. 10.1172/JCI62839 23281408 PMC3533272

[B15] Voors A. A. Dorhout B. van der Meer P. (2013). The Potential Role of Valsartan + AHU377 ( LCZ696 ) in the Treatment of Heart Failure. Expert Opin. Investig. Drugs. 22 (8), 1041–1047. 10.1517/13543784.2013.797963 23663006

[B16] White H. D. Norris R. M. Brown M. A. Brandt P. W. Whitlock R. M. Wild C. J. (1987). Left Ventricular End-Systolic Volume as the Major Determinant of Survival after Recovery from Myocardial Infarction. Circulation. 76 (1), 44–51. 10.1161/01.cir.76.1.44 3594774

[B17] Xie M. Burchfield J. S. Hill J. A. (2013). Pathological Ventricular Remodeling: Therapies: Part 2 of 2. Circulation. 128 (9), 1021–1030. 10.1161/CIRCULATIONAHA.113.001879 23979628 PMC3829603

